# Supplementary material for: Vaginal Microbial Network Analysis Reveals Novel Taxa Relationships among Adolescent and Young Women with Incident Sexually Transmitted Infection Compared with Those Remaining Persistently Negative over a 30-Month Period
Source: Microorganisms. 2023 Aug 8;11(8):2035. doi: 10.3390/microorganisms11082035 (PMC10459434; doi:10.3390/microorganisms11082035)
Supplement: Supplementary file 1 [file microorganisms-11-02035-s001.zip › microorganisms-2467329-supplementary.pdf]

microorganisms

Supplementary Materials

Table S1. Number of tests conducted, infections detected, and treatments by study visit.

Time point	Baseline, N=436<br>n (%)	6-Months, N=424<br>n (%)	12 Months, N=397<br>n (%)	18 Months, N=399<br>n (%)	30 Months, N=396<br>n (%)
Bacterial vaginosis (BV) Tested for	436	424	395	398	395
BV Detected	49 (11.2)	39 (9.2)	57 (14.4)	56 (14.1)	88 (22.3)
BV Treated	48 (98.0)	37 (94.9)	55 (96.5)	56 (100)	79 (89.8)
Number of BV not detected at any prior visit	--	19 (48.7)	29 (50.9)	31 (55.4)	39 (49.4)
Number of incident BV		20	28	25	49
Chlamydia trachomatis (CT) Tests Conducted	436		395		395
CT Detected	27 (6.2)		26 (6.6)		47 (11.9)
CT Treated	25 (92.6)		26 (100)		44 (93.6)
Number of CT infections detected at a prior visit	---		6 (23.1)		9 (6.4)
Neisseria gonorrhoeae (NG) Tests Conducted	436		395		395
NG Detected	6 (1.4)		12 (3.0)		8 (2.0)
NG Treated	6 (100)		12 (100)		6 (75.0)
Number of NG infections detected at a prior visit	---		2 (16.7)		1 (16.7)
Trichomonas vaginalis (TV) Tests Conducted	436		395		395
TV Detected	13 (3.0)		13 (3.3)		20 (5.1)
TV Treated	13 (100)		12 (92.3)		15 (75.0)
Number of TV infections detected at a prior visit	---		0 (0.0)		2 (10.0)
Composite Sexually Transmitted Infection (STI) Tests Conducted	436		395		395
STI Detected	43 (9.9)		46 (11.7)		64 (16.2)
STI Treated	41 (95.3)		46		57
Any[1] STI detected at any prior visit	---		13		22
Total number incident composite STI (i.e., infections not preceded by <u>any</u> STI at a prior visit) [2]			33		42
Order of incident STI and/or incident BV	Prevalent				
Persistent negative for both (no prior infection)	359 (82.3)		263 (88.9)		180 (81.1)
Incident STI (no BV prior) [2]	28 (6.4)		24 (8.1)		17 (7.7)
BV before incident STI	34 (7.8)		6 (2.0)		14 (6.3)
Incident STI and Incident BV	15 (3.4)		3 (1.0)		11 (5.0)
CT only (no TV or NG)					
Incident CT (no BV prior)			12		9
BV before incident CT			3		8
Incident CT and Incident BV			2		7
TV only (no CT or NG)					
Incident TV (no BV prior)			7		6
BV before incident TV			2		3
Incident TV and Incident BV					1
NG only (no CT or TV)					
Incident NG (no BV prior)			3		
BV before incident NG					1
Incident NG and Incident BV					1

[1] If a participant had TV at baseline, and then CT at 12 months or 30 months, this would not be included in analysis of incident STI. Incident STI is defined as no prior STI infection with any etiology (CT, NG, or TV). This is because the antimicrobial treatment for the first incident STI would affect VMB composition for assessment of subsequent incident STI. [2] There were n=2 participants with incident STI infections (no prior BV) and n=1 persistently negative participant that was excluded from analyses due to having <5,000 total sequence read counts.

Table S2. Characteristics of give participants with incident Sexually transmitted infection and incident Bacterial vaginosis detected at the same study visit and participants with incident *Chlamydia trachomatis* in the absence of other STIs and BV.

	Incident *C. trachomatis* in the absence of other STIs and BV, N=21<br>n (%)	Incident *T. vaginalis* in the absence of other STIs and BV, N=13<br>n (%)
At Baseline		
Randomization status		
Control arm	11 (52.4)	4 (30.8)
Cup arm	10 (47.6)	9 (69.2)
Median Age in years		
<16.9 years	9 (42.9)	7 (53.9)
>=16.9 years	12 (57.1)	6 (46.1)
Socioeconomic status score		
Highest quintiles	13 (61.9)	9 (69.2)
Lowest quintiles	8 (38.1)	4 (30.8)
Water, sanitation, and hygiene score		
Higher	9 (42.9)	7 (53.9)
Lower	12 (57.1)	6 (46.1)
Sexually active		
No	13 (61.9)	8 (61.5)
Yes	8 (38.1)	5 (38.5)
Had transactional sex		
No	19 (90.5)	10 (76.9)
Yes	2 (9.5)	3 (23.1)
Experienced coerced sex		
No	13 (61.9)	9 (69.2)
Yes	8 (38.1)	4 (30.8)
Has a boyfriend		
No	19 (90.5)	12 (92.3)
Yes	2 (9.5)	1 (7.7)
Vaginal Community State Type (CST)		
CST-I (*L. crispatus* dominated)	9 (45.0)	3 (27.3)
CST-III (*L. iners* dominated)	9 (45.0)	6 (54.5)
CST-IV (mixed)	2 (10.0)	2 (18.2)
At time of Incident STI		
Time of incident STI		
12 months	12 (57.1)	7 (53.9)
30 months	9 (42.9)	6 (46.1)
Median Age in Years		
<18.8 years	13 (61.9)	7 (53.9)
>=18.8 years	8 (38.1)	6 (46.1)
Socioeconomic status score		
Median or higher	13 (65.0)	10 (76.9)
Below median	7 (35.0)	3 (23.1)
Ever Sexually active		
No	6 (30.0)	6 (50.0)
Yes	14 (70.0)	6 (50.0)
Had transactional sex		
No	16 (80.0)	11 (84.6)
Yes	4 (20.0)	2 (15.4)
Experienced coerced sex		
No	15 (75.0)	12 (92.3)
Yes	5 (25.0)	1 (7.7)
Has a boyfriend		
No	11 (55.0)	10 (76.9)
Yes	9 (45.0)	3 (23.1)
Vaginal Community State Type		
CST-I (*L. crispatus* dominated)	1 (4.8)	3 (27.3)
CST-III (*L. iners* dominated)	19 (90.5)	1 (9.1)
CST-IV (mixed)	1 (4.8)	7 (63.6)

*Not all cells sum to N due to missing data.

Table S3. Presence and mean relative abundance (RA) of 54 taxa by outcome status*.

	Persistently STI and BV Negative, N=179		Incident STI and BV Negative, N=39		Incident STI and BV Before STI, N=20		Incident STI and BV at Same Time, N=14	
	Present (%)	Mean RA (%)	Present (%)	Mean RA (%)	Present (%)	Mean RA (%)	Present (%)	Mean RA (%)
Lactobacillus crispatus	124 (69.3)	37.8	20 (51.3)	14.7	5 (25.0)	3.17	2 (14.3)	0.01
Lactobacillus iners	117 (65.4)	28.1	33 (84.6)	38.5	17 (85.0)	40.2	12 (85.7)	16.1
Gardnerella vaginalis	94 (52.5)	4.94	27 (69.2)	6.73	18 (90.0)	9.73	12 (85.7)	13.9
Lactobacillus spp.	125 (69.8)	4.10	21 (53.9)	1.20	8 (40.0)	0.23	4 (28.6)	0.66
Sneathia sanguinegens	30 (16.8)	2.02	16 (41.0)	5.11	12 (60.0)	6.81	10 (71.4)	17.4
Lactobacillus jensenii	28 (15.6)	2.36	7 (18.0)	0.35	2 (10.0)	0.62	0 (0)	0
Bacteria, not otherwise specified	171 (95.5)	1.05	39 (100)	3.30	17 (85.0)	3.90	13 (92.9)	0.75
Prevotella timonensis	94 (52.5)	1.11	24 (61.5)	2.01	16 (80.0)	1.52	13 (92.9)	4.42
Finegoldia magna	154 (86.0)	1.13	35 (89.7)	1.55	17 (85.0)	1.70	14 (100)	4.15
Anaerococcus prevotii	127 (70.9)	1.38	26 (66.7)	1.36	15 (75.0)	1.33	11 (78.6)	1.96
Ureaplasma urealyticum	90 (50.3)	1.19	29 (74.4)	1.34	12 (60.0)	0.92	7 (50.0)	0.37
Megasphaera spp.	20 (11.2)	0.50	5 (12.8)	0.89	9 (45.0)	4.18	9 (64.3)	5.45
Prevotella bivia	59 (33.0)	1.24	18 (46.2)	1.08	9 (45.0)	0.95	8 (57.1)	1.72
Peptoniphilus gorbachii	119 (66.5)	0.83	25 (64.1)	0.92	15 (75.0)	1.37	13 (92.9)	2.58
Lactobacillus reuteri	97 (54.2)	1.00	7 (18.0)	0.20	7 (35.0)	0.45	1 (7.1)	0.01
Fannyhessea vaginae (Atopobium)	24 (13.4)	0.54	12 (30.8)	0.46	13 (65.0)	1.24	10 (71.4)	3.42
Gemella haemolysans/gemella asaccharolytica	18 (10.1)	0.26	9 (23.1)	2.16	11 (55.0)	2.28	9 (64.3)	1.87
BVAB1	8 (4.5)	0.27	3 (7.7)	0.38	6 (30.0)	3.04	2 (14.3)	1.57
Lactobacillus gasseri	42 (23.5)	0.57	9 (23.1)	1.77	2 (10.0)	0.06	1 (7.1)	0.10
Veillonella spp.	27 (15.1)	0.51	8 (20.5)	1.16	9 (45.0)	0.97	3 (21.4)	0.44
Costridiales Family XI	105 (58.7)	0.73	24 (61.5)	0.27	11 (55.0)	0.62	10 (71.4)	0.64
Prevotella spp.	57 (31.8)	0.30	16 (41.0)	0.43	10 (50.0)	1.89	12 (85.7)	2.56
Dialister succinatiphilus	38 (21.3)	0.26	6 (15.4)	0.11	12 (60.0)	1.44	11 (78.6)	3.38
Aerococcus christensenii	20 (11.2)	0.16	11 (28.2)	0.68	13 (65.0)	1.49	9 (64.3)	1.60
Prevotella corporis	66 (36.9)	0.52	12 (30.8)	0.19	6 (30.0)	0.34	4 (28.6)	0.41
Streptococcus anginosus	27 (15.1)	0.32	7 (18.0)	0.83	3 (15.0)	0.13	3 (21.4)	2.65
Prevotella disiens	50 (27.9)	0.23	18 (46.2)	1.59	11 (55.0)	0.29	8 (57.1)	0.57
Streptococcus oralis	11 (6.2)	0.78	4 (10.3)	0.07	1 (5.0)	0.75	0 (0)	0
Dialister propionicifaciens	73 (40.8)	0.32	17 (43.6)	0.57	11 (55.0)	0.18	9 (64.3)	0.54
Prevotella amnii	5 (2.8)	0.15	3 (7.7)	0.04	7 (35.0)	1.33	5 (35.7)	2.43
Sneathia amnii	7 (3.9)	0.15	5 (12.8)	0.8	2 (10.0)	1.38	1 (7.1)	0.03
Firmicutes spp.	37 (20.7)	0.23	15 (38.5)	0.70	5 (25.0)	0.39	5 (35.7)	0.21
Mycoplasma hominis	13 (7.3)	0.10	14 (35.9)	1.04	8 (40.0)	0.27	7 (50.0)	1.15
Staphylococcus hominis	75 (41.9)	0.41	9 (23.1)	0.17	4 (20.0)	0.03	0 (0)	0
Porphyromonas asaccharolytica	56 (31.3)	0.30	17 (43.6)	0.54	10 (50.0)	0	9 (64.3)	0.40
Brassicibacter spp.	39 (21.8)	0.17	15 (38.5)	1.10	2 (10.0)	0.2	3 (21.4)	0.08
Prevotella colorans	43 (24.0)	0.24	12 (30.8)	0.10	9 (45.0)	0.99	7 (50.0)	0.44
Fusobacterium equinum	9 (5.0)	0.31	3 (7.7)	0.15	2 (10.0)	0.06	3 (21.4)	0.37
Dialister micraerophilus	50 (27.9)	0.19	20 (51.3)	0.53	13 (65.0)	0.42	13 (92.9)	0.65
Anaerococcus spp.	37 (20.7)	0.22	14 (35.9)	0.23	2 (10.0)	0.26	2 (14.3)	0.83
Porphyromonas bennonis	79 (44.1)	0.23	18 (46.2)	0.33	7 (35.0)	0.21	8 (57.1)	0.22
Prevotella buccalis	60 (33.5)	0.26	14 (35.9)	0.14	7 (35.0)	0.14	9 (64.3)	0.85
Corynebacterium coyleae	62 (34.6)	0.21	14 (35.9)	0.41	4 (20.0)	0.03	3 (21.4)	0.38
Peptostreptococcus anaerobius	38 (21.2)	0.13	19 (48.7)	0.61	12 (60.0)	0.44	5 (35.7)	0.12
Lactobacillus coleohominis	49 (27.4)	0.19	13 (33.3)	0.21	1 (5.0)	0.003	2 (14.3)	0.07
Prevotella melaninogenica	7 (3.9)	0.26	7 (18.0)	0.85	4 (20.0)	0.07	3 (21.4)	0.34
Haemophilus influenzae	6 (3.4)	0.31	1 (2.6)	0.15	1 (5.0)	0.003	0 (0)	0
Alloprevotella rava	28 (15.6)	0.34	4 (10.3)	0.63	0	0	4 (28.6)	0.24
Clostridiales	62 (34.6)	0.20	14 (35.9)	0.17	11 (55.0)	0.17	7 (50.0)	0.17
Fusobacterium nucleatum	19 (10.6)	0.26	5 (12.8)	0.84	6 (30.0)	0.36	5 (35.7)	0.26
BVAB2	4 (2.2)	0.02	3 (7.7)	0.02	7 (35.0)	0.74	8 (57.1)	1.18
Porphyromonas somerae	40 (22.4)	0.16	14 (35.9)	0.05	5 (25.0)	0.23	5 (35.7)	0.23
Prevotellaceae bacterium	35 (19.6)	0.21	6 (15.4)	0.12	1 (5.0)	0.01	0 (0)	0
Anaerococcus hydrogenalis	44 (24.6)	0.13	9 (23.1)	0.16	5 (25.0)	0.07	3 (21.4)	0.05

* Taxa presence and relative abundances are reported at time of infection, or at time of sampling if persistently negative.

Table S4. Distribution of Network properties by Outcome: Persistently negative for STI and BV and BV prior to incident STI.

Network properties	Persistently Negative, No STI and No BV N=179	BV Prior to Incident STI N=20	Absolute Difference
**Largest Connected Component (LCC)**			
Relative LCC size	0.438	0.438	0
Clustering coefficient	0.320	0.0	0.320
Modularity	0.309	0.441	0.132
Positive edge percentage	78.9	78.6	0.376
Edge density	0.209	0.154	0.055
Natural connectivity	0.101	0.096	0.004
Vertex connectivity	1.00	1.00	0
Edge connectivity	1.00	1.00	0
Average dissimilarity	0.687	0.691	0.005
Average path length	2.33	3.63	1.30
**Whole network**			
Number of components	9	8	1
Clustering coefficient	0.309	0	0.309
Modularity	0.561	0.727	0.166
Positive edge percentage	88.2	76.0	12.2
Edge density	0.069	0.050	0.018
Natural connectivity	0.039	0.038	0.002
**Degree Centrality [1]**			
Fannyhesea vaginae (Atopobium)	0.194	0	0.167
Sneathia amnii	0.161	0	0.161
Gemella haemolysans/ Gemella asaccharolytica	0.129	0	0.129
Firmicutes spp.	0	0.097	0.097
Prevotella bivia	0	0.065	0.065
Prevotella spp.	0	0.065	0.065
Prevotella colorans	0	0.065	0.065
Porphyromonas somerae	0	0.065	0.065
Porphyromonas asaccharolytica	0	0.065	0.065
Lactobacillus spp.	0.065	0	0.065
**Betweenness Centrality [2]**			
Megasphaera	0	0.628	0.628
Porphyromonas somerae	0	0.513	0.513
Lactobacillus jensenii	0	0.462	0.462
Firmicutes spp.	0	0.410	0.410
Fannyhesea vaginae (Atopobium)	0.385	0	0.385
Prevotella amnii	0.192	0.474	0.282
Gemella haemolysans/ Gemella asaccharolytica	0.218	0	0.218
Sneathia amnii	0.205	0	0.205
Streptococcus oralis	0.179	0	0.179
Sneathia sanguinegens	0	0.154	0.154
**Closeness Centrality [3]**			
Fannyhesea vaginae (Atopobium)	0.704	0	0.704
Sneathia amnii	0.671	0	0.671
Gemella haemolysans/ Gemella asaccharolytica	0.593	0	0.593
Veillonella spp.	0.498	0	0.498
Lactobacillus spp.	0.478	0	0.478
Porphyromonas somerae	0	0.450	0.450
Firmicutes spp.	0	0.444	0.444
Gardnerella vaginalis	0.443	0	0.443
Dialister succinatiphilus	0.426	0	0.426
Prevotella bivia	0	0.423	0.423
**Eigenvector Centrality [4]**			
Fannyhesea vaginae (Atopobium)	1.00	0	1.00
Sneathia amnii	0.850	0	0.850
Prevotella bivia	0	0.546	0.546

Gemella haemolysans/ Gemella asaccharolytica	0.517	0	0.517
Porphyromonas somerae.	0	0.493	0.493
Megasphaera	0.493	0.958	0.464
Prevotella spp.	0	0.439	0.439
Prevotella colorans	0	0.405	0.405
Gardnerella vaginalis	0.311	0	0.311
Lactobacillus spp.	0.304	0	0.304

[1] Jaccard P-value =0.263. [2]Jaccard P-value =0.088. [3]Jaccard P-value =0.263. [4]Jaccard P-value =0.461.
